# Dosimetry verification of three‐dimensional printed polylactic acid template‐guided precision ^125^I seed implantation for lung cancer using a desktop three‐dimensional printer

**DOI:** 10.1002/acm2.13419

**Published:** 2021-09-06

**Authors:** Xiaoyan Han, Shu Fang, Rui Sheng, Yi Wang, Jinhua Zhou, Jiong Wang

**Affiliations:** ^1^ Department of Geriatric Respiratory and Critical Care Anhui Geriatric Institute the First Affiliated Hospital of Anhui Medical University Hefei City China; ^2^ School of Biomedical Engineering Anhui Medical University Hefei City China; ^3^ Chaohu Clinical Medical College Anhui Medical University Chao Hu City China

**Keywords:** 3D printing, brachytherapy dosimetry, brachytherapy, ^125^I seeds, PLA template

## Abstract

**Introduction:**

The purpose of this study was to verify the effectiveness of polylactic acid (PLA) template puncture route planning by comparing preoperative and postoperative dosimetry using computerized tomography (CT)‐guided implantation of ^125^I radioactive seeds.

**Methods:**

A total of 28 patients who underwent ^125^I seed implantation between January 2018 and June 2019 were selected for the statistical study of seed dosimetry. All patients received preoperative treatment planning system (TPS) planning, of which 13 patients in the experimental 3D template group underwent intraoperative puncture and implantation using the PLA template planning route. The other 15 patients in the traditional control group underwent intraoperative puncture and implantation using CT images for guidance. By calculating the dose‐volume histogram, preoperative and postoperative D90 values and postoperative V90 values were compared between the two groups.

**Results:**

The mean D90 values in the template group before and after surgery were 136.06 ± 7.10 and 134.72 ± 7.85 Gy, respectively. There was no statistically significant difference. The preoperative and postoperative mean D90 values in the traditional group were 132.97 ± 8.04 and 126.06 ± 9.19 Gy, respectively, which were statistically significantly different. The mean postoperative V90 values in the template and traditional groups were 93.80 ± 1.34% and 88.42 ± 6.55 %, respectively, showing a statistically significant difference.

**Conclusions:**

The preoperative TPS plan for the experimental group guided by the PLA template was almost the same as that for the final guided particle implantation. The dose parameters in the experimental group were also better than those in the traditional group, making the use of the presented PLA template more efficient for clinical applications.

## 1 INTRODUCTION

Lung cancer currently occupies the first place in the incidence and mortality of malignant tumors in China, with the age group of >60 years old accounting for 40% of lung cancer patients. This proportion tends to gradually increase as society ages. For most elderly patients diagnosed with advanced lung cancer for the first time, in addition to the benefits of radiotherapy and chemotherapy, palliative treatment is effective in relieving symptoms and improving quality of life. Since the beginning of this century, minimally invasive interventional therapy for solid tumors has achieved local efficacy. Brachytherapy with ^125^I radioactive seeds has been favored by more doctors and patients as an internal therapy that has the advantage of precise dose, few adverse reactions, and minimally invasive intervention.^1^ ^125^I particle emits gamma rays with 27–35 keV energy and has a half‐life of about 60 days. They can be used with satisfactory efficacy to continuously irradiate the target volume to kill tumor cells, especially for the treatment of various solid tumors.^2^

3D printing is an emerging technology based on digital model files that uses special adhesive materials to construct objects using layer‐by‐layer printing. In particular, medical 3D printing can be utilized to create surgical models, puncture surgical guides, implants of various materials, and even tissues and organs.^3^ Chinese oncology experts have applied 3D‐printed coplanar and non‐coplanar templates to radioactive seed implantation for a variety of malignant tumors, greatly improving its accuracy. However, current product technology has a high standard and price, which is not conducive to being systematically deployed on a large scale. The present study used a desktop‐grade 3D printer and polylactic acid (PLA) as a raw material to design and print implanted radioactive ^125^I seed surgical guides for clinical use in lung cancer and evaluated the dose differences when compared to traditional methods of ^125^I seed implantation.^4^

## 2 METHODS AND MATERIALS

### 2.1 Patient selection

A total of 28 pathologically confirmed lung cancer patients were enrolled in the study at the Department of Respiratory and Critical Care of the First Affiliated Hospital of Anhui Medical University between January 2018 and June 2019. There were 21 males and seven females (mean age: 66 years; range: 33–78 years; Table 1). None of the included patients had surgical indications or refused surgery. All patients signed up for local radioactive particle implantation treatment. They had no vital organ dysfunction, no vital organ metastasis, no abnormalities in blood routine and biochemical and coagulation function, and had a Karnofsky score >60. Patients with severe organ dysfunction, coagulation disorders, acute and chronic infection, and psychiatric history were excluded from the present study. The study was reviewed and approved by the hospital's ethics committee and all patients provided their written informed consent.

**TABLE 1:** Treatment characteristics before ^125^I seeds implantation (*n* = 28)

**No**.	**Gender**	**Age**	**Diagnosis**	**^a^** **Stage**	**Tumor volume (cm^3^)**	**Seed activity (mCi)**	**Seed numbers/PD (Gy)**	**D90 (Gy)**	**Seeds implant**
1	Female	65	Left lung adenocarcinoma	T4N1M1 IV	46	0.6	56/110	133.6	PLA template‐guided
2	Female	63	Right lung squamous carcinoma	T3N0M0 IIb	48	0.6	57/110	135.8	PLA template‐guided
3	Male	61	Double lung squamous carcinoma	T2N0M1 IV	29	0.6	36/110	121.9	PLA template‐guided
4	Male	73	Left lung squamous carcinoma	T2N0M1 IV	53	0.6	77/110	133.0	PLA template‐guided
5	Male	68	Left lung adenocarcinoma	T3N3M1 IV	60	0.6	63/110	136.0	PLA template‐guided
6	Male	73	Left lung squamous carcinoma	T3N1M0 IIIA	78	0.6	120/110	146.6	PLA template‐guided
7	Male	69	Left lung squamous carcinoma	T2N3M0 IIIB	72	0.6	103/110	144.3	PLA template‐guided
8	Male	63	Left lung adenocarcinoma	T4N0M1 IV	52	0.6	88/110	137.5	PLA template‐guided
9	Male	73	Right lung squamous carcinoma	T4N3M0 III C	47	0.6	50/110	125.6	PLA template‐guided
10	Male	33	Left lung adenocarcinoma	T2N1M1 IV	73	0.6	106/110	147.0	PLA template‐guided
11	Male	69	Right lung adenocarcinoma	T3N2M1 IV	69	0.6	99/110	138.5	PLA template‐guided
12	Female	64	Left lung adenocarcinoma	T3N2M1 IV	80	0.6	88/110	138.6	PLA template‐guided
13	Male	69	Left lung squamous carcinoma	T3N3M1 IV	39	0.6	52/110	132.1	PLA template‐guided
14	Male	63	Left lung adenocarcinoma	T4N0M1 IV	41	0.6	51/110	134.4	PLA template‐guided
15	Female	73	Left lung squamous carcinoma	T2N1M1 IV	48	0.6	101/110	135.6	CT guided
16	Male	64	Left lung squamous carcinoma	T2N3M1 IV	29	0.6	46/110	122.6	CT guided
17	Female	65	Left lung adenocarcinoma	T4N1M1 IV	29	0.6	55/110	139.0	CT guided
18	Male	68	Left lung squamous carcinoma	T1N3M0 IIIB	26	0.6	41/110	131.0	CT guided
19	Male	78	Left lung squamous carcinoma	T2N3M1 IV	31	0.6	39/110	124.0	CT guided
20	Male	64	Right lung squamous carcinoma	T3N3M1 IV	45	0.6	76/110	128.0	CT guided
21	Male	69	Left lung squamous carcinoma	T4N2M0 IIIB	43	0.6	55/110	133.1	CT guided
22	Male	71	Left lung squamous carcinoma	T3N3M0 IIIC	77	0.6	103/110	139.4	CT guided
23	Male	62	Left lung squamous carcinoma	T3N1M1 IV	98	0.6	158/110	145.6	CT guided
24	Female	64	Left lung adenocarcinoma	T2N2M0 IIIA	63	0.6	101/110	134.9	CT guided
25	Male	75	Left lung adenocarcinoma	T4N0M0 IIIA	69	0.6	83/110	126.0	CT guided
26	Male	60	Right lung adenocarcinoma	T2N2M0 IIIA	49	0.6	39/110	131.0	CT guided
27	Male	64	Right lung squamous carcinoma	T3N3M0 IIIC	88	0.6	110/110	146.4	CT guided
28	Female	71	Right lung squamous carcinoma	T4N0M1 IV	98	0.6	111/110	135.0	CT guided

Abbreviations: CT, computerized tomography; PLA, polylactic acid.

^a^ The stage of tumor was done by TNM classification of malignant tumors in 2017.

### 2.2 Equipment and software

Treatment planning system (TPS; Beijing Tianhang Colin Technology Development Co., Ltd., Beijing City, China); Template design software: E‐3D digital medical modeling and design platform (Research Center for Digital Health and Virtual Reality, Central South University, Changsha City, China); Puncture needle: 18G (batch number: SFDA Certified No. 20173156872); Computerized tomography (CT): SOMATOM Force; 3D printer: Type A8S (Aurora Erwo Technology Co., Ltd., Shenzhen City, China); ^125^I seeds (batch number: SFDA Certified No. H20041695, activity of ^125^I is 0.5–0.7 mCi, Ningbo Jun'an Pharmaceutical Technology Co., Ltd., Ningbo City, China); and negative pressure vacuum pad.

### 2.3 Preoperative positioning

**FIGURE 1:**
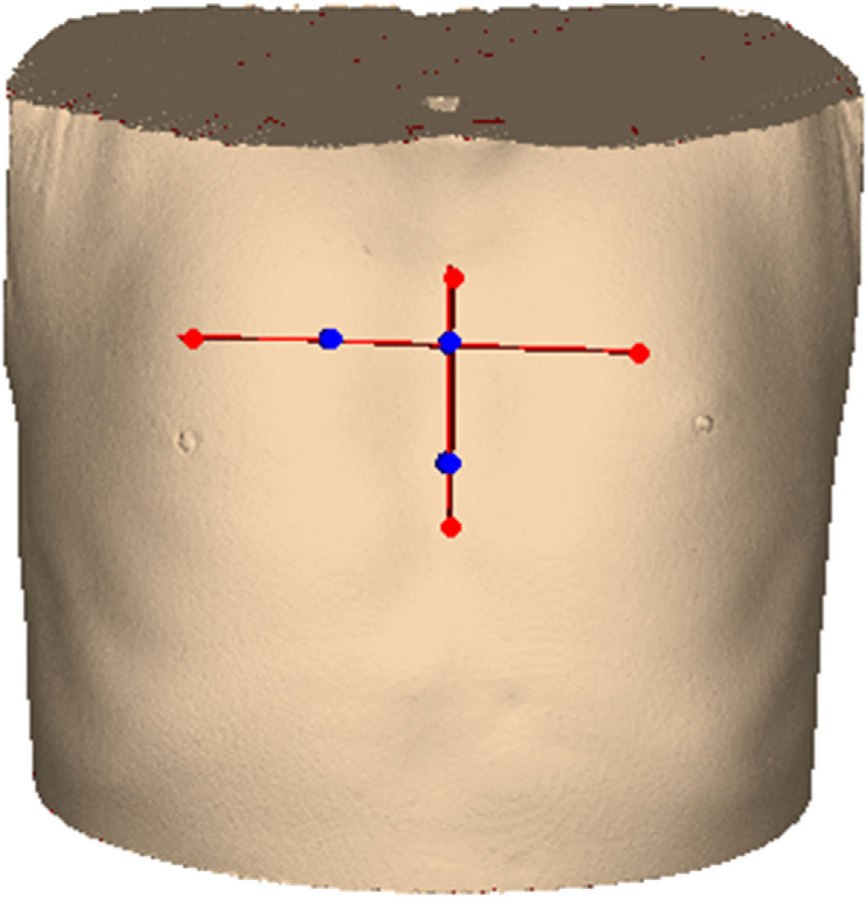
Position marker: Place three blue hemispherical objects according to the laser line

First, the negative pressure vacuum pad was placed on the preoperative spiral CT flatbed and the CT positioning laser line was then turned on. Next, the positioning marking lines of the negative pressure vacuum pad, patient's skin surface, and CT laser were overlapped. A positioning ball with a 1 cm diameter was placed at the intersection of the laser line and surface midline. Two additional positioning balls were placed on the CT laser positioning line perpendicular to each other 3 cm laterally and 3 cm vertically in relation to the area where the lesion was located. The three positioning balls formed a triangle to facilitate the final positioning of the patient and to reduce the size of the PLA template (Figure 1).

### 2.4 Lesion mapping

The Digital Imaging and Communications in Medicine‐formatted image data for the patients' preoperative chest lesions served as the data source. In order to restore the authenticity of the image data, multi‐slice spiral CT with a high tissue image resolution (over 32 layers) was selected. The scan was performed with a 1 mm thickness standard in order to facilitate tissue segmentation using E‐3D software. If the lesion was located close to the blood vessels, enhanced chest CT was recommended. If it was accompanied by atelectasis, a positron emission tomography (PET)‐CT examination was recommended to ensure that the tumor target was correctly delineated. After accessing data using E‐3D software, the lasso tool was used in the coronal and sagittal planes to obtain a 3D contour figure drawing of lesions. The multi‐layer editing tools were used to contour cross‐sectional lesions. The manual modification was used to ensure the correctness of the tumor target delineation, improving the focal range of fidelity and ensuring the accuracy of puncture. For radioactive particle implantation, it is necessary to expand the outline of the lesion outward by 1 cm to obtain the clinical target area. Sparse and narrow burrs can be omitted in the process of composition, while dense and short burrs need to be outlined.^5^

### 2.5 3D modeling

**FIGURE 2:**
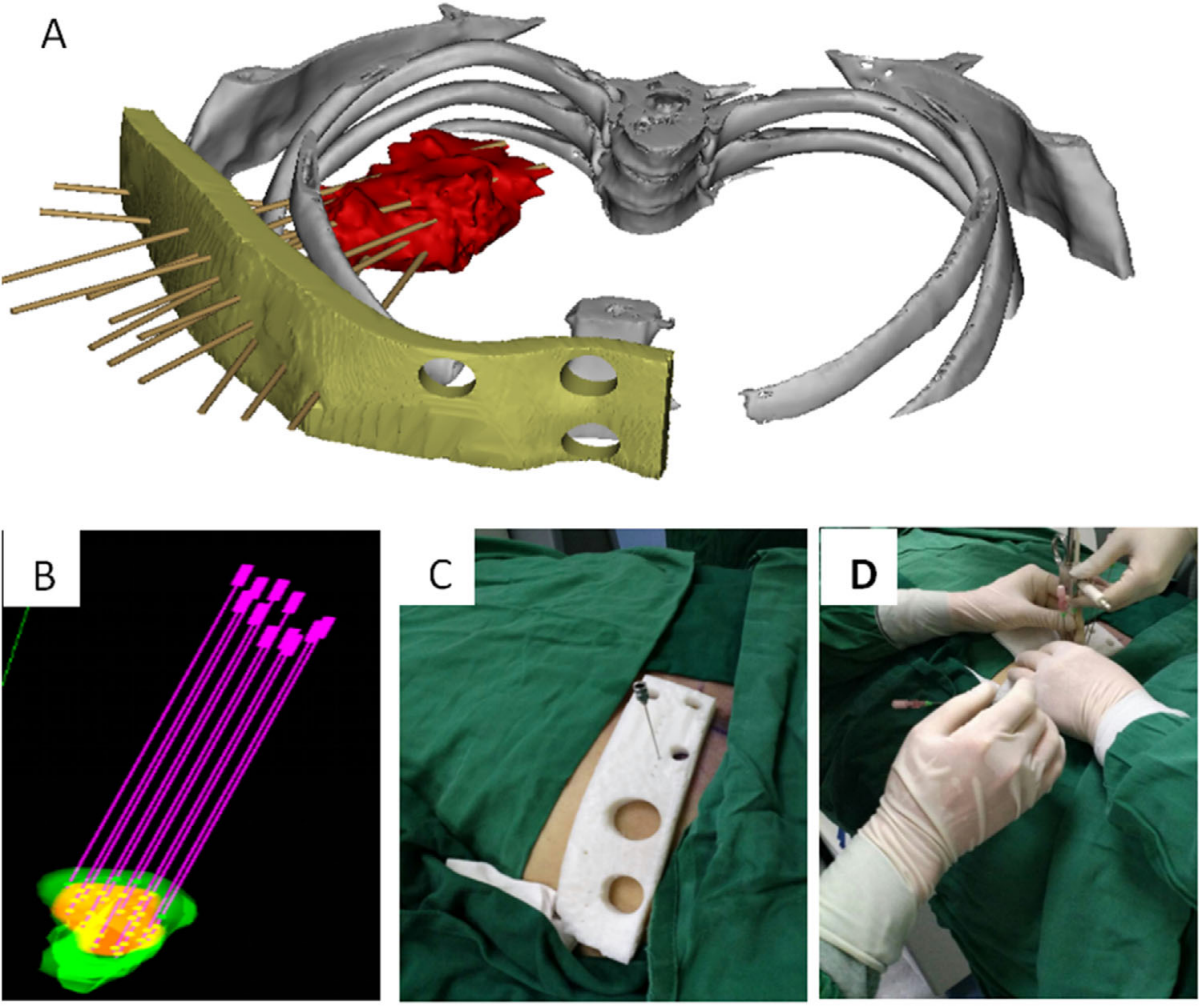
(a) Three‐dimensional reconstruction of the lesion, (b) Radioactive seed distribution within the lesion, (c) Surgical guide plate made using polylactic acid (PLA), and (d) Radioactive seed implantation was performed using PLA surgical guide plate

A threshold segmentation tool was used to mark the thoracic structure and its periphery with different color masks. Then, morphological operation tools were used to expand and edit the thoracic surface (set the thickness to 10 mm). A thoracic surface mask was obtained using a Boolean operation for subtraction. Finally, 3D reconstruction was performed on the thoracic structure, body surface mask, and diseased portion (Figure 2a).

### 2.6 Template needle track design

Since the size of the puncture needle used in the operation was 18G and the diameter was 1.2 mm, the inner diameter of the template needle track was expanded to 1.5 mm when considering the error margin of the 3D printer. The design of the needle track was strictly based on the treatment plan, including the number and location of the seed arrangement. Using the cylinder design, the needle track was transformed from the invisible to the visible state, and then the cylinder and the chest mask were subtracted using Boolean calculation. The hollow cylinder appeared on the chest mask, which was the actual needle track. Finally, the chest mask was converted to stereolithography format and input into a 3D printer for printing (Figure 2b).

### 2.7 Mark template printing

The stereolithography‐formatted template data were imported into the Chinese version of the slicing software CURA15.06 (Ultimaker Holding B. V. Utrecht, Netherlands). The printing speed was adjusted to 45 mm/s, while the filling degree was set to 100%. The PLA particle implantation guidance template with needle track information and reset mark was printed using PLA as the material using the desktop‐level Aurora A8S 3D printer. It was then sent to the hospital's disinfection supply center for sterilization.

### 2.8 Template reset

**FIGURE 3:**
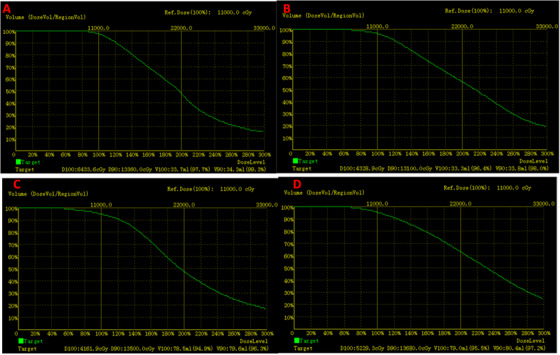
(a) Pre‐treatment dose‐volume histogram (DVH) graph for the templated group, (b) Post‐treatment DVH graph for the templated group, (c) Pre‐treatment DVH graph for the traditional group, and (d) Post‐treatment DVH graph for the traditional group

The PLA template was reset using the spiral CT positioning laser line, where the binding and triangle positioning marks were overlapped using three positioning balls on the body surface. Intraoperative optimization and postoperative verification were performed according to TPS after all needle transplants were completed. Isodose distribution curve and dose‐volume histogram (DVH) were also obtained (Figures 2c,d and  3).

### 2.9 Statistical analysis

Statistical analysis was performed using SPSS version 21.0 statistical software (SPSS Inc., Chicago, Illinois, USA). In this analysis, independent samples *t*‐tests and the Wilcoxon test were used to analyze the difference in D90 values before and after the operation and V90 values between the two groups. *p* < 0.05 was considered statistically significant.

## 3 RESULTS

This study has successfully explored a set of procedures for making a PLA radioactive seed implantation surgical guide plate using a desktop 3D printer. Compared to other guides, this PLA guide plate has a lower cost and faster printing speed. Its safety and effectiveness have been successfully verified clinically.

The preoperative planning and postoperative dosimetric parameters in 28 patients showed no statistically significant differences in preoperative and postoperative D90 values in the PLA group (*p* > 0.05), while preoperative and postoperative D90 values in the traditional group were statistically significantly different (*p* < 0.05; Table 2). The postoperative V90 values for the two groups were statistically significantly different (*p* < 0.05; Table 3). These results showed that implantation of radioactive seeds under the guidance of the PLA guide plate printed on a desktop 3D printer fulfilled the preoperative TPS treatment plan well.

**TABLE 2:** Pre‐ and post‐operation D90 values of two groups

	**Preoperation D90 (Gy)**	**Postoperation D90 (Gy)**	***t/Z***	***p***
PLA template‑guided group	136.06 ± 7.10	134.72 ± 7.85	–0.552	0.581
Traditional group	132.97 ± 8.04	126.06 ± 9.19	–2.275	0.023

**Abbreviation: PLA,:** polylactic acid.

**TABLE 3:** Postoperation V90 values of the two groups

	**PLA template‑guided group**	**Traditional group**	***t***	***p***
V90	93.80 ± 1.34	88.42 ± 6.55	3.011	0.009

**Abbreviation: PLA,:** polylactic acid.

## 4 DISCUSSION

In recent years, Chinese experts have started using the operation guide plate created using 3D technology to guide the implantation of radioactive seeds to treat solid tumors. Their efforts have achieved good therapeutic effects and have been highly praised by the majority of clinicians. However, the current surgical guide plate is expensive, its design and printing are controlled by the manufacturer and it is not included in China's medical security system, which is difficult for patients to accept and restricts its clinical application. In the present study, the PLA surgical guide plate was designed using medical image processing software and printed with a tabletop 3D printer to guide the implantation of radioactive seeds for the treatment of lung cancer.^6^ CT‐guided technology is under the guidance of CT, using needle, catheter, guidewire, and other instruments to directly reach the lesion site, for biopsy or treatment. There are two kinds of puncture guidance techniques: Conventional CT guided (CCT) and real‐time CT fluoroscopy. Conventional CT is used in order to reduce the absorbed radiation dose. Compared to the traditional method, the preoperative TPS treatment plan was realized, indicating a good clinical application effect. These results were consistent with research by Zhang et al.^8^ Desktop 3D printing is a revolutionary tool that has become popular around the world in recent years due to its small size. Solid objects can be printed using an ordinary office desktop. PLA and acrylonitrile butadiene styrene are typical raw materials required for 3D printing, which have the benefits of low cost and easy maintenance.^7^

At present, E‐3D software has powerful functions and is free for clinicians to use. It is very simple and convenient to design a surgical guide board. The output files for the design are not encrypted and can be printed by the user. Compared to CT‐guided puncture surgery, the surgical guide plate does not need to rely on the operator to complete the surgery. The following advantages characterize the application of the surgical guide plate: 1. PLA is cheap, and the production cycle is short, so patients do not need to wait too long, which reduces the risk of a sudden change in lesions in a short period of time; 2. The research cost is greatly reduced so that more energy can be used to focus on dosimetry research. Under the guidance of the preoperative TPS plan in the present study, E‐3D digital medical modeling software developed by the Central South University of China was used to design the surgical guide plate. The results showed that there was no statistically significant difference in D90 values in the PLA surgical guide group before and after surgery (*p* > 0.05). There was a statistically significant difference in D90 values in the traditional group before and after surgery (*p* < 0.05), indicating that it was difficult for the traditional treatment to meet the dose requirements of the preoperative plan. This is because the free‐hand puncture implantation of radioactive seeds cannot guarantee that the spatial positioning in the tumor is arranged according to the preoperative plan. Therefore, the dose error before and after the operation is difficult to accurately control. The error in D90 values in the PLA surgical guide plate group before and after surgery was 4 Gy, while the error in the traditional group was 18 Gy. The significant difference between the two groups indicated that the use of a 3D‐printed surgical guide plate can significantly reduce the dose error, which is consistent with Hongtao et al.^8^ research results. However, they used medical resin to make the surgical guide plate, which is too expensive to become a popular option. In addition, the surgical guide made using PLA achieves the same effect at one‐tenth of the cost. Liaw et al.^9^ study confirmed that large preoperative and postoperative D90 errors seriously affect clinical efficacy, while preoperative and postoperative dose fluctuations of the surgical guide plate are lower and have better repeatability. The difference in postoperative V90 values between the PLA surgical guide plate group and the traditional group was statistically significant (*p* < 0.05), which demonstrated the unique advantage of the surgical guide plate in the dose distribution. At present, this surgical operation mainly depends on the experience of the operator, and different operators will cause greater dose differences. However, consistent results were obtained even though the operation in the present study was performed by different doctors. Therefore, the use of a surgical guide plate can significantly reduce dose errors and ensure clinical efficacy.^10^, ^11^, ^12^

We have made some improvements to the printing method and accumulated experience based on prior work. First, the parameter setting for 3D printing is particularly critical. For example, the printing filling degree directly determines the weight of the surgical guide plate, which in turn affects the degree of its collapse on the body surface. When the surgical guide plate is placed on the body surface, it is easy to make the skin sag, which leads to needle direction deviation and affects dosage accuracy. However, a printing filling degree that is too low affects printing accuracy. Therefore, a reasonable printing filling degree is an important research focus. Second, CT scan layer thickness and image resolution have a direct impact on the recognition accuracy of the lesion contour using medical image processing software. The thinner the layer is, the higher the resolution, which means that the tumor edge will be marked more accurately, also affecting the accuracy of the direct image dose.^13^, ^14^ In this study, the scanning parameter included 1 mm thickness, while the target area was delineated under the lung window to ensure a precise dose design. Third, printing speed settings affect printing quality, such as the smoothness of the inner wall of the puncture needle. Therefore, setting appropriate parameters can lay a good foundation for successful construction and accurate printing of the 3D radioactive seed implantation guide plate. During the study, we found that the PLA surgical guide plate also had some shortcomings in clinical application. Compared to the current mainstream medical resin materials, the PLA surgical guide plate is not compatible with the marking pigment. The information marked on the surface of the guide plate is easy to follow. Thus, information such as needle track number and depth cannot be printed on the surface. Therefore, an assistant is needed for oral guidance with the help of a computer, where the puncture depth of each needle track must be recorded before the puncture. In addition, the number of seeds was inconsistent before and after surgery, which may be due to the fact that lung tumors are vulnerable to the influence of breathing movement.^15^ Although patients were provided with breathing and posture training, it was not enough to completely eliminate the influence of movement, leading to inevitable errors in practical operation. Based on the results presented in this work, large tumors need to be investigated in further studies.

## 5 CONCLUSION

In summary, this study explored and established a new method of implantation of PLA radioactive seeds for lung cancer into a surgical guide plate designed based on medical image processing software and printed using a desktop 3D printer. This method has demonstrated a good application effect in clinical use and is worthy of further clinical promotion and application.

## AUTHOR CONTRIBUTIONS

**Xiaoyan Han** and **Shu Fang** designed and wrote the paper, and contributed equally to this work;Ideas, developmentor design of methodology; **Rui Sheng**: Programming and provision of study materials; **Yi Wang**: Acquisition of the financial support for the project leading to this publication; **Jinhua Zhou**: Management and coordination responsibility for the research activity planning and execution, acquisition of the financial support for the project leading to this publication; and **Jiong Wang**: Oversight and leadership responsibility for the research activity planning and execution, including mentorship external to the core team.

## CONFLICT OF INTEREST

The authors have no conflicts of interest to declare.
